# Malignant character of an ossified posterior longitudinal ligament in a hyperflexion injury: A case report

**DOI:** 10.3892/mi.2024.172

**Published:** 2024-06-26

**Authors:** George Fotakopoulos, Vasiliki Epameinondas Georgakopoulou, Nikolaos Trakas, Pagona Sklapani, Alexandros G. Brotis

**Affiliations:** 1 Department of Neurosurgery, General University Hospital of Larissa, 41221 Larissa, Greece; 2 Department of Pathophysiology, National and Kapodistrian University of Athens, 11527 Athens, Greece; 3 Department of Biochemistry, Sismanogleio Hospital, 15126 Athens, Greece

**Keywords:** ectopic ossification of the posterior longitudinal ligament, ankylosing spondylitis, cervical spine ossification of the posterior longitudinal ligament, cervical osteophyte

## Abstract

The present study reports the case of a 50-year-old obese male with ankylosing spondylitis, Scheuermann's kyphosis. The patient was asymptomatic concerning the ectopic ossification of the posterior longitudinal ligament (OPLL) at the cervical spine; he developed quadriparesis and respiratory insufficiency following minor head trauma. Even though trauma to the cervical spine in patients with OPLL is common, to the best of our knowledge, this is the first reported case of an extensive osteophyte with a lethal outcome after syncope. In rare occasions, it may be present with syncope and potentially lethal outcomes, particularly when precipitated by trauma. Therefore, the management of OPLL with significant canal stenosis should not be unnecessarily delayed.

## Introduction

The ectopic ossification of the posterior longitudinal ligament (OPLL) represents a localized form of skeletal hyperostosis. Its annual incidence is as high as 3 and 1.3% for Asian and non-Asian populations, respectively (1). It frequently involves the cervical and thoracic spine to a lesser extent.

The nature of this chronic disease is generally benign. OPLL is commonly associated with significant stenosis of the spinal canal. The majority of patients present with myelopathy in the fifth and sixth decades of life. During the disease, up to 17% of the cases require some form of assistance in the activities of daily living. Patients are at an increased risk of developing quadriparesis following cervical spine trauma, reaching as high as 15% (1).

The present study describes the case of an asymptomatic patient with OPLL who developed quadriparesis and respiratory insufficiency following minor head trauma. The patient succumbed shortly afterwards due to a respiratory infection.

## Case report

**Figure 1:**
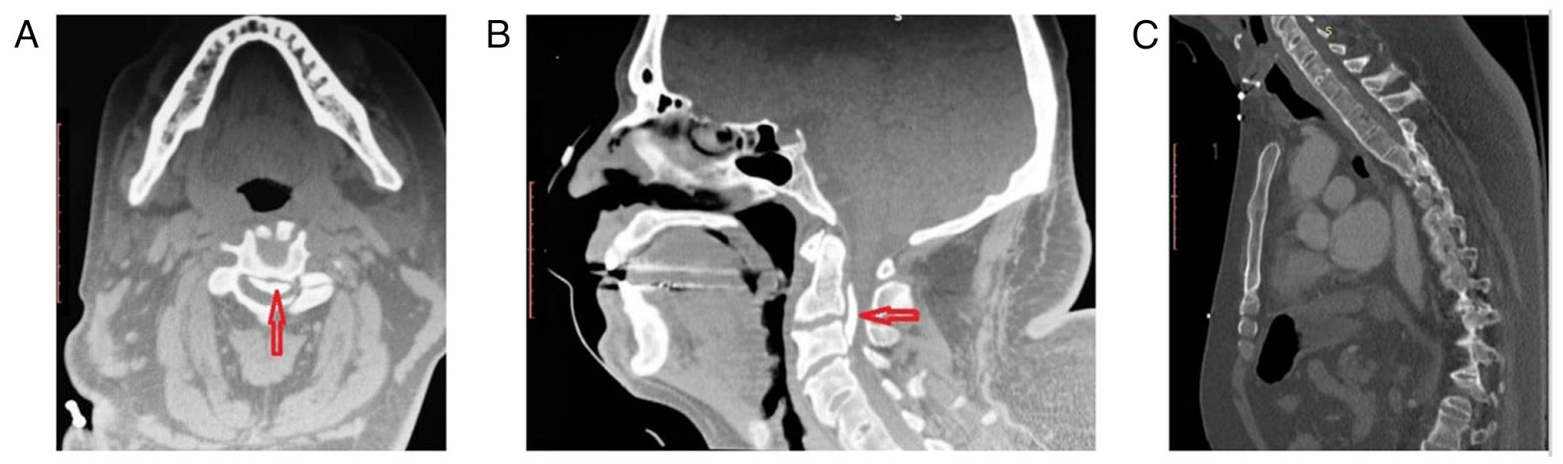
(A) A pre-operative CT scan of the cervical spine (sagittal view) and (B) axial view of a 50-year-old obese male with ectopic ossification of the posterior longitudinal ligament (red arrows); (C) pre-operative CT scan of the cervical and thoracic spine (axial view) illustrating ankylosing spondylitis and Scheuermann's kyphosis. CT, computed tomography.

During hospitalization for a urinary tract infection, a 50-year-old obese male with ankylosing spondylitis (AS), Scheuermann's kyphosis (KS) and a body mass index of 42.4, lost consciousness and suffered a minor head injury at the occipital region. The immediate clinical examination revealed that the patient was hemodynamically stable (85 bpm and 123/85 mmHg) and febrile (39˚C) (from a urinary tract infection with pyospheres; a urine culture was positive for *Escherichia. coli*); he had sufficient respiration (SAO_2_, 97%). After 1-2 min, the patient regained consciousness but exhibit no contraction (0/5) in all key muscle groups and sensory paralysis during the neurological examination (ASIA A). A head computed tomography (CT) scan revealed no evidence of intracranial hemorrhage or other intracranial pathology and his Glasgow Coma Scale (GCS) score was 15/15. However, the CT scan of the cervical spine revealed an OPLL associated with severe spinal canal stenosis (canal diameter, 4.18 mm) and extensive anterior ankylosis (Fig. 1). After 30 min, the patient developed respiratory distress and was intubated using fiberoptic technology.

**Figure 2:**
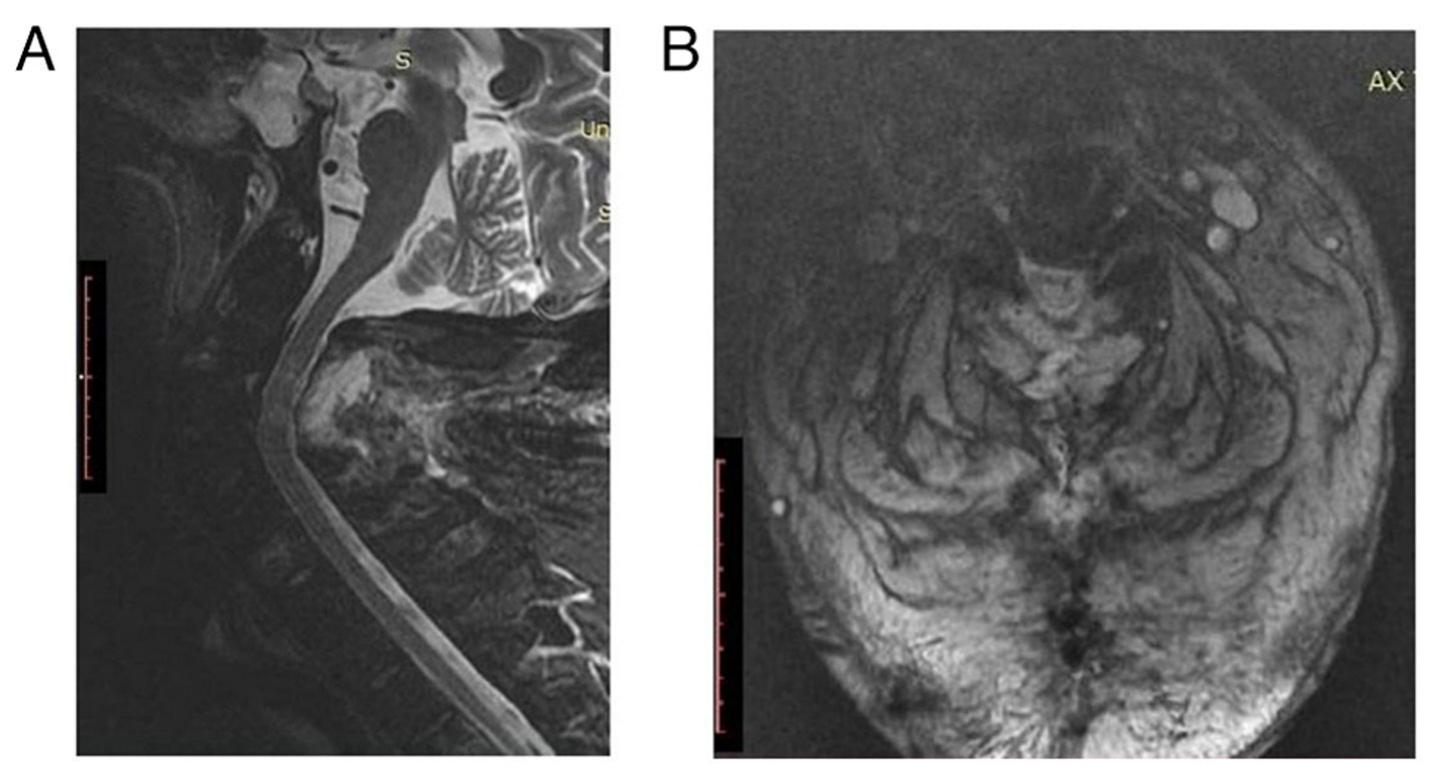
(A) Sagittal view and (B) axial view of a post-operative magnetic resonance imaging scan of a cervical spine documented the adequacy of decompression.

At that time, the patient was transferred to the University Hospital of Larissa (Larissa, Greece) with a cervical rigid collar. In the operating room, he underwent posterior cervical spine decompression with a laminectomy extending from C2 to C4. Following surgery, the patient was awakened and transferred to the intensive care unit for further cardiopulmonary support. The patient remained quadriplegic, with a minor improvement in the deltoid muscles (1/5). The post-operative magnetic resonance imaging scan documented the adequacy of decompression (Fig. 2). Additionally, it revealed a spinal cord with an increased signal intensity, compatible with edema. On the 6th day, the patient suffered a cardiac arrest, which he survived following half an hour of cardiopulmonary resuscitation. Moreover, a cardiac pacemaker was inserted to avoid future episodes. On the 11th day, the patient developed an acute abdomen following gastrostomy tube placement, for which he underwent an exploratory laparotomy. On the following day, the patient suffered a massive pulmonary embolism despite adequate anticoagulation and finally succumbed.

## Discussion

Even though trauma to the cervical spine in patients with OPLL is common, to the best of our knowledge, this is the first reported case of an extensive osteophyte with a lethal outcome after a syncope (1,2). The most common presentation of OPLL is cervical myelopathy from the chronic narrowing of the spinal canal, followed by quadriparesis precipitated by trauma (1-4).

The association between the OPLL and syncope is not clear. Several pathogenetic mechanisms could be implicated. A vasogenic origin cannot be excluded following the compression of the anterior spinal arteries and blood stagnation in the vertebrobasilar system. It is unclear whether vertigo caused by vascular changes at the vertebrobasilar circulation causes syncope (5). Moreover, a neurogenic mechanism could be considered after an acute compression of the vagal and glossopharyngeal nuclei in the higher cervical region with parasympathetic over-discharge (5). Finally, the patient may have simply lost consciousness due to a hypotensive episode during an acute urinary tract infection or a latent arrhythmia.

In the present study, pre-operative imaging revealed an OPLL at the C1, C2, and C3 vertebral levels, corresponding to a continuous ossification based on the relevant classification pattern (1). Of note, two additional findings are worth noting, including a marked canal narrowing at the levels corresponding to the canal compromise and ankylosis of the subaxial spine compatible with AS (1,3). The co-existence of AS, KS and OPLL is infrequent, but both have been associated with human leukocyte antigen variants (1,3).

Symptomatic cases are usually treated surgically (1-3). Anterior procedures aim to remove the ossified ligament and directly decompress the spinal canal, but with a high risk of unintended durotomy (1-3). On the other hand, posterior approaches decompress the spinal canal indirectly (1,2). In the case presented herein, the posterior approach was preferred based on the level of the lesion and the curvature of the cervical spine. The K-line, a virtual line between the midpoints of the anteroposterior canal diameter at C2 and C7, fell behind the osteophyte, necessitating a posterior approach (6).

Studies have reported the association between obesity and hyperostosis situations resembling OPLL and diffuse idiopathic skeletal hyperostosis, where the mechanism of enormous cumulatively formed osteophytes remains unclear (7,8). However, the underlying mechanism may be connected with insulin-resistant states, and the surplus adipose tissue via mechanical, hormonal, and cytokine factors leads to bone upregulation (9,10).

In conclusion, OPLL is a rare disease that usually manifests with cervical myelopathy. In rare occasions, it may present with syncope and potentially lethal outcomes, particularly when precipitated by trauma. Therefore, the management of OPLL with marked canal stenosis should not be unnecessarily delayed. Further studies are required for the validation of the findings presented herein.

## Data Availability

The datasets used and/or analyzed during the current study are available from the corresponding author on reasonable request.
